# Portable 3D Gait Analysis Assessment in MTT Treat Chronic Ankle Instability: A Retrospective Study

**DOI:** 10.1155/2021/6098978

**Published:** 2021-06-07

**Authors:** Yujuan Song, Sibai Xu, Yanqiu Dai, Jun Jia, Hebin Liu, Zhenjing Li

**Affiliations:** ^1^TCM Department, Shenzhen Longhua District Central Hospital, China; ^2^High-Level Medical Team Project Work Group, Shenzhen Longhua District Central Hospital, China

## Abstract

**Purpose:**

Retrospective analysis of the effect of portable 3D gait analysis as an innovative evaluation method in the treatment with MTT on chronic ankle instability patient.

**Methods:**

From January 1, 2019, to December 31, 2019, 56 cases of chronic ankle instability (CAI) were extracted from the medical record system of Shenzhen Longhua District Central Hospital. All the patients of 56 cases accepted the medical training therapy (MTT). As outcome parameters, the alterations of the Cumberland ankle instability tool (CAIT), foot and ankle ability measure (FAAM), were used before the treatment and after treatment; meanwhile, the portable apparatus 3D gait analysis was used to measure the gait parameters.

**Conclusion:**

The results showed only ankle angle parameters *Y*-axis, maximum dorsiflexion during support period (°) had a significant difference, and the *p* value is 0.039. Meanwhile, the CAIT, FAAM, and most 3D gait analysis data had no significant difference. This particular statistical difference shows that CAI can be measured scientifically and objectively, although most measurement parameters have no change. These results make further reveal that the CAI patients are suffering with dynamic abnormality of ankle motion angle; this also provides us with a measurable and systematic evaluation reference plan for CAI treatment in the future.

## 1. Introduction

Ankle sprain is one of the most common musculoskeletal injuries in physical activities. Patients with ankle sprain will suffer from decreased ankle stability and recurrent sprain several months and years after the initial ankle injury, which is the characteristic of chronic ankle instability (CAI) [1]. About 40% of the patients with first-time ankle sprain developed CAI symptoms during the 12-month follow-up, repeated sprains caused by CAI can lead to injuries of soft tissues such as ligaments and tendons of the ankle joint, and severe ankle sprains may induce ankle osteoarthritis [2]. Therefore, it is necessary to carry out correct and effective intervention or treatment. At present, the main nonsurgical intervention methods of rehabilitation medicine for CAI are exercise therapy, ankle protectors, intramuscular patches, etc. Medical training therapy (MTT) is a comprehensive medical sports rehabilitation technology, which is mainly used in sports injuries, postoperative rehabilitation, bone and joint diseases, limb motor dysfunction, pain, and other fields. Our group retrospectively analyzed the medical records of MTT of CAI, in order to further evaluate its clinical efficacy.

## 2. Clinical Data

### 2.1. Research Object

Patients with CAI who accepted MTT in the Rehabilitation Department, or Traditional Chinese Medicine Department of the Shenzhen Longhua District Central Hospital from January 1, 2019, to December 31, 2019, were extracted from the medical record system of Shenzhen Longhua District Central Hospital. Finally, according to the inclusion and exclusion criteria, a total of 56 subjects were determined.

### 2.2. Diagnostic Criteria

The diagnostic criteria of CAI were formulated with reference to the screening criteria of CAI proposed by the International Ankle Consortium in 2014 [3]: (a) a history of at least 1 significant ankle sprain, the initial sprain must have occurred at least 12 months prior to enrollment; (b) a history of the previously injured ankle joint “giving away” and/or recurrent sprain and/or “feeling of instability”; (c) Ankle Instability Instrument (AII): answer “yes” to at least 5 yes/no questions; Cumberland ankle instability (CAIT): <24; Identification of Functional Ankle Instability (IdFAI) > 11; foot and ankle ability measure (FAAM): ADL scale < 90%, sport scale < 80%; Foot and Ankle Outcome Score (FAOS): <75% in 3 or more categories.

### 2.3. Inclusion Criteria

The inclusion criteria were as follows: (a) the diagnostic criteria above are met; (b) medical records are complete, the MTT prescription is entered at least once a week, and there are assessment scales before and after treatment; (c) the length of treatment is at least 2 weeks; (d) the contact information can meet follow-up criteria; (e) participation is voluntary, and informed consent has been signed.

### 2.4. Exclusion Criteria

The exclusion criteria were as follows: (a) patients with a history of lower limb fracture or surgery, or other musculoskeletal diseases; (b) patients with acute injury of lower limbs within three months; (c) the length of treatment is less than 2 weeks; (d) incomplete medical records and lack of important diagnosis and treatment information; (e) lack of contact information and inability to cooperate with follow-up.

## 3. Treatment

**Figure 1:**
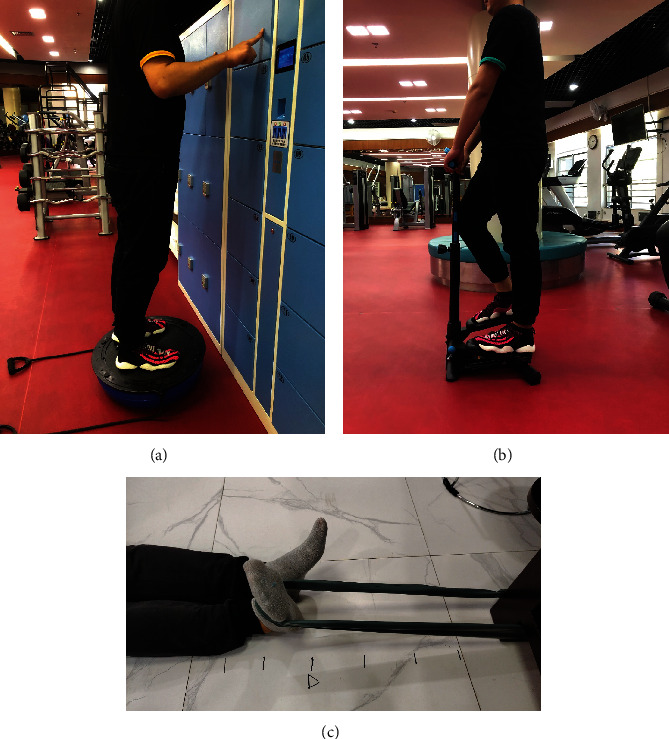
The 3D gait analysis: (a) the exercise training of the ankle, (b) the balance training, and (c) the resistance training.

Before the first treatment, all the patients accepted the examination and evaluation of the safety of MTT by rehabilitation physicians and physiotherapists. The treatments occurred 3 times per week for 8 weeks under the supervision of physiotherapists. The procedures of the treatment are as follows:

- The resistance training of the ankle [4]. The training was carried out with rubber-resistance bands (DOMYOS), according to different elastic forces, it was set to four levels: red → green → blue → orange. The patients progressed to next color level biweekly. (1) Calculation of the resisting force of the training: measured the stretching distance of rubber-resistance band, and marked the point of 70% of the maximum stretching length of rubber-resistance bands on the floor. (2) The patients sat on the floor and kept their knees straight and neutral. The rubber-resistance band was folded in half; one end was fixed, and the other end was wrapped around the involved foot. (3) Stretched the rubber-resistance band to the marked point only by the involved foot, then performed the training in 4 directions: inversion, eversion, plantar flexion, and dorsiflexion. Repeated 10 times in every direction for one group, 3 groups per treatment [5]
- The balance training of the ankle. The training was carried out with the balance training board (DOMYOS Balance Board). With the patients in an upright standing position and fingers supporting the wall, the involved foot stood in the center of the balance training board and kept balance for 40 s, then changed direction after 10 s rest. Repeated 5 times for one group, 5 groups per treatment [6]
- The exercise training of the ankle. The training was carried out with stair steppers (DECATHLON MS100 Stepper). Placed feet on the stair stepper to perform alternate pedaling actions. Repeated 50 times for one group, 3 groups per treatment. See Figures 1(a)–1(c)

## 4. Outcomes

We set 3D gait analysis data as primary outcome, and the secondary outcome was the Cumberland ankle instability tool (CAIT), foot and ankle ability measure (FAAM).

## 5. Evaluation

### 5.1. 3D Gait Analysis

**Figure 2:**
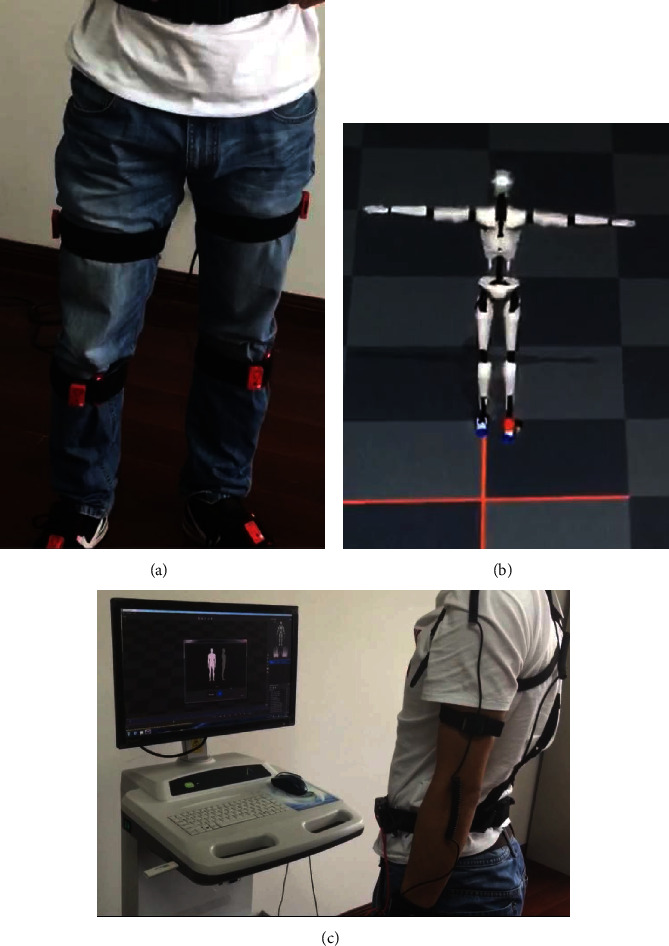
The 3D gait analysis: (a) wearable biomarkers, (b) computer software modeling, and (c) movement and model action matching.

Before and after treatment, we applied 3D gait analysis (Real Gait, NeuCogic Medical Co., Ltd.) to measure the gait parameters. Temporal and spatial parameters, such as stride frequency, step length, walking speed, stride length, and max ankle angles at vertical, horizontal, and coronal planes, were recorded with Portable Apparatus Real Gait [7]. Before the test, the operator calibrated the parameters of the instrument and explained the procedure of the test to the patients. After they fully understood the evaluation process, we begin to conduct a gait analysis test and get gait-related parameters [8]. For the 3D gait analysis, please see Figures 2(a)–2(c).

### 5.2. Cumberland Ankle Instability Tool (CAIT)

Before and after treatment, CAIT questionnaire was used to evaluate the pain and stability of the ankle in patients' daily life, such as walking, running, going downstairs, standing on one leg, and turning sharply.

### 5.3. Foot and Ankle Ability Measure (FAAM)

FAAM consists of two subscales, among which 21 items of the FAAM-ADL scale are related to activities of daily living, and 8 items of the FAAM-sport scale are related to sports.

## 6. Statistical Methods

The data was presented as mean and standard deviations (SD). The data was analyzed by an independent statistician with IBM SPSS Statistics 21.0. A two-sided *p* value of less than 0.05 was defined as statistical significance. Paired *t*-test was used in the data comparison between before and after treatment.

## 7. Results

The baseline data was listed in the following; it indicated the basis information of the patients. Please see Table 1.

The CAIT and FAAM data comparison results showed there was no significant difference between before and after treatment. Please see Table 2.

**Figure 3:**
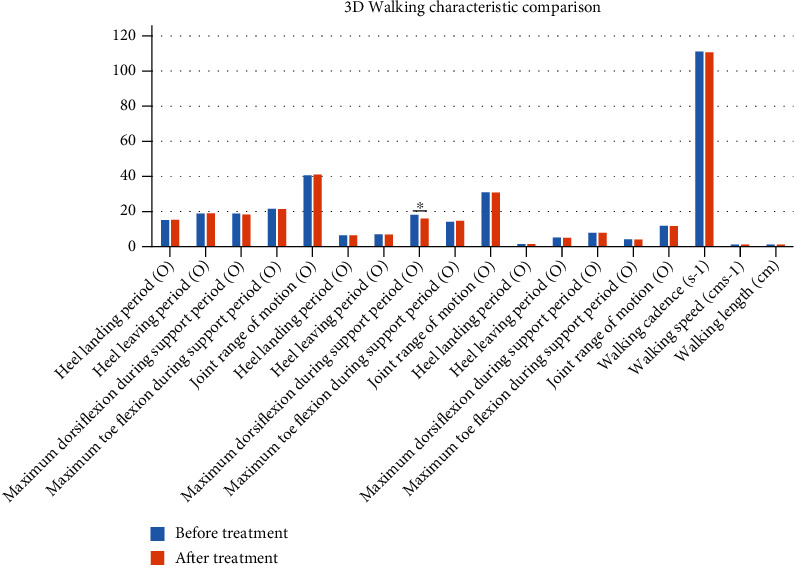
3D walking characteristic mathematical absolute value comparison between before and after treatment. ^∗^sig.<0.05.

The 3D walking characteristic data comparison showed most results had no significant difference, while the “maximum dorsiflexion during support period” had the *p* value which was 0.039. Please see Figure 3 and Table 3.

## 8. Discussion

The ankle is one of the main joints bearing gravity and ground reaction force; the feet bear the ground reaction force of 1.5 times of body weight when walking; moreover, when running, it can reach 2-3 times of body weight. Therefore, ankle sprain is one of the most common sports injuries, accounting for about 30% of them [9–11]. After ankle sprain, there may be a variety of aftereffects, including ankle instability, pain, and weakness. In view of this phenomenon, scientists have done a lot of related researches [12]. Hertel [7] defined the phenomenon of repeated instability and multiple sprains of the ankle as CAI. Recent studies have shown that the occurrence of chronic ankle instability is related to ligament injury, insufficient muscle strength, delayed muscle reaction time, and weakened ankle proprioception [13].

Medical training therapy (MTT) is one form of nonsurgical treatment advocated by guidelines, it is a sport rehabilitation system based on science and evidence, and it helps the impaired structure and function to recover comprehensively by different sport training [14]. MTT has the characteristics of planning, systematicness, and initiative; the core elements of MTT are joint flexibility, coordination, endurance, and muscle strength. MTT focuses on active exercise and combines passive exercise to encourage patients' subjective active participation. Therefore, it not only improves patients' body function but also emphasizes patients' comprehensive physical and mental rehabilitation [15, 16]. In this retrospective study, we also found that MTT can relieve the CAI patients' ankle pain and improve ankle instability.

At present, the research on CAI mainly focuses on the biomechanical mechanisms such as muscle strength, balance function, and posture stability. As gait analysis helps to describe the characteristics of the gait of CAI patients, and quantitative analysis of the biomechanical changes of the ankle, we apply it to the treatment evaluation of CAI [17, 18]. The kinematic characteristics of gait can be reflected by the following parameters: step length, pace, pace frequency, step width, step angle, gait cycle and the percentage of each phase in the total cycle, the movement angle and angular velocity of the three joints of lower limbs in three planes, etc. Monaghan et al. [19] analyzed the gait of patients with CAI 100 ms before landing and 200 ms after landing, and the results showed that patients with CAI had more varus than the normal group during normal walking, and the swing speed of the heel swing period was significantly faster than that of the normal group.

In this study, the CAIT and FAAM data showed no significant difference; meanwhile, the 3D gait analysis assessment showed nearly the same results; however, uniquely, the data of ankle angle parameters *Y*-axis and maximum dorsiflexion during support period (°) showed a significant difference when *p* value is 0.039. This particular statistical difference shows that CAI can be measured scientifically and objectively, although most measurement parameters have no change. These results make further reveal that the CAI patients are suffering with dynamic abnormality of ankle motion angle; this also provides us with a measurable and systematic evaluation reference plan for CAI treatment in the future.

## Figures and Tables

**Table 1:** Baseline data.

Items	Values
*N* (case)	56
Mean age (y)	34.05
SD age	12.72
Female/male	31/25
Left/right	17/39
CAIT mean (score)	23.53
CAIT SD	6.04
FAAM-ADL mean (score)	95.88
FAAM-ADL SD	14.15
FAAM-sports mean (score)	72.90
FAAM-sports SD	10.22

**Table 2:** CAIT and FAAM score comparison between before and after treatment.

		Before treatment	After treatment	.sig
CAIT	*N* (case)	56	56	1
	Mean (score)	23.53	24.06	0.816
	SD	6.04	10.80	
	Max (score)	27	27	
	Minimum (score)	21	20	

FAAM-ADL	Mean (score)	95.88	95.91	0.902
	SD	14.15	18.55	

FAAM-sports	Mean (score)	72.90	73.03	0.561
	SD	10.22	12.16	

**Table 3:** 3D walking characteristic comparison between before and after treatment.

		Before treatment mean (SD)	After treatment mean (SD)	.sig
Ankle angle parameters *X*-axis	Heel landing period (°)	15.16 (3.71)	15.21 (4.18)	>0.05
	Heel leaving period (°)	-18.91 (4.66)	-19.02 (3.80)	>0.05
	Maximum dorsiflexion during support period (°)	18.80 (4.61)	18.19 (6.77)	>0.05
	Maximum toe flexion during support period (°)	-21.47 (3.10)	-21.45 (4.46)	>0.05
	Joint range of motion (°)	40.55 (11.85)	41.08 (8.02)	>0.05

Ankle angle parameters *Y*-axis	Heel landing period (°)	6.38 (1.03)	6.46 (1.90)	>0.05
	Heel leaving period (°)	7.05 (1.17)	6.92 (2.06)	>0.05
	Maximum dorsiflexion during support period (°)	18.14 (12.99)	15.96 (7.00)^∗^	0.039
	Maximum toe flexion during support period (°)	-14.18 (4.95)	-14.70 (3.11)	>0.05
	Joint range of motion (°)	30.95 (8.06)	30.82 (6.90)	>0.05

Ankle angle parameters *Z*-axis	Heel landing period (°)	-1.51 (0.27)	-1.49 (0.35)	>0.05
	Heel leaving period (°)	5.22 (4.28)	5.06 (3.72)	>0.05
	Maximum dorsiflexion during support period (°)	7.82 (4.08)	7.86 (4.22)	>0.05
	Maximum toe flexion during support period (°)	4.09 (2.80)	4.01 (3.06)	>0.05
	Joint range of motion (°)	11.90 (6.67)	11.70 (5.49)	>0.05

Walking space characteristics	Walking cadence (s^−1^)	111.05 (5.04)	110.62 (6.85)	>0.05
	Walking speed (cms^−1^)	1.10 (0.09)	1.10 (0.20)	>0.05
	Walking length (cm)	1.11 (0.02)	1.11 (0.04)	>0.05

## Data Availability

The study data used to support the findings of this study are currently under embargo while the research findings are commercialized. Requests for data, 24 months after publication of this article, will be considered by the corresponding author.

## References

[1] Delahunt E., Coughlan G. F., Caulfield B., Nightingale E. J., Lin C. W. C., Hiller C. E. (2010). Inclusion criteria when investigating insufficiencies in chronic ankle instability. *Medicine and Science in Sports and Exercise*.

[2] Doherty C., Bleakley C., Hertel J., Caulfield B., Ryan J., Delahunt E. (2016). Recovery from a first-time lateral ankle sprain and the predictors of chronic ankle instability. *The American Journal of Sports Medicine*.

[3] Gribble P., Delahunt E., Bleakley C. M. (2014). Selection criteria for patients with chronic ankle instability in controlled research: a position statement of the International Ankle Consortium. *Journal of Athletic Training*.

[4] Hale S. A., Hertel J., Olmsted-Kramer L. C. (2007). The effect of a 4-week comprehensive rehabilitation program on postural control and lower extremity function in individuals with chronic ankle instability. *The Journal of Orthopaedic and Sports Physical Therapy*.

[5] Hall E. A., Docherty C. L., Simon J., Kingma J. J., Klossner J. C. (2015). Strength-training protocols to improve deficits in participants with chronic ankle instability: a randomized controlled trial. *Journal of Athletic Training*.

[6] Hall E. A., Chomistek A. K., Kingma J. J., Docherty C. L. (2018). Balance- and strength-training protocols to improve chronic ankle instability deficits, part I: assessing clinical outcome measures. *Journal of Athletic Training*.

[7] Hertel J. (2000). Functional instability following lateral ankle sprain. *Sports Medicine*.

[8] Donovan L., Hertel J. (2012). A new paradigm for rehabilitation of patients with chronic ankle instability. *The Physician and Sportsmedicine*.

[9] Holmes A., Delahunt E. (2009). Treatment of common deficits associated with chronic ankle instability. *Sports Medicine*.

[10] Cain M. S., Ban R. J., Chen Y. P., Geil M. D., Goerger B. M., Linens S. W. (2020). Four-week ankle-rehabilitation programs in adolescent athletes with chronic ankle instability. *Journal of Athletic Training*.

[11] Wenning M., Gehring D., Lange T. (2021). Clinical evaluation of manual stress testing, stress ultrasound and 3D stress MRI in chronic mechanical ankle instability. *BMC Musculoskeletal Disorders*.

[12] Cho J. H., Lee D. H., Song H. K., Bang J. Y., Lee K. T., Park Y. U. (2016). Value of stress ultrasound for the diagnosis of chronic ankle instability compared to manual anterior drawer test, stress radiography, magnetic resonance imaging, and arthroscopy. *Knee Surgery, Sports Traumatology, Arthroscopy*.

[13] Lee K. T., Park Y. U., Jegal H., Park J. W., Choi J. P., Kim J. S. (2014). New method of diagnosis for chronic ankle instability: comparison of manual anterior drawer test, stress radiography and stress ultrasound. *Knee Surgery, Sports Traumatology, Arthroscopy*.

[14] Lin C. I., Khajooei M., Engel T. (2021). The effect of chronic ankle instability on muscle activations in lower extremities. *PLoS One*.

[15] DeJong A. F., Mangum L. C., Hertel J. (2019). Gluteus medius activity during gait is altered in individuals with chronic ankle instability: an ultrasound imaging study. *Gait & Posture*.

[16] Feger M. A., Donovan L., Hart J. M., Hertel J. (2015). Lower extremity muscle activation in patients with or without chronic ankle instability during walking. *Journal of Athletic Training*.

[17] Fuerst P., Gollhofer A., Wenning M., Gehring D. (2021). People with chronic ankle instability benefit from brace application in highly dynamic change of direction movements. *Journal of Foot and Ankle Research*.

[18] Smith B. I., Docherty C. L., Simon J., Klossner J., Schrader J. (2012). Ankle strength and force sense after a progressive, 6-week strength-training program in people with functional ankle instability. *Journal of Athletic Training*.

[19] Monaghan K., Delahunt E., Caulfield B. (2006). Ankle function during gait in patients with chronic ankle instability compared to controls. *Clinical Biomechanics*.

